# Supplementary material for: Complexome profiling of the Chlamydomonas psb28 mutant reveals TEF5 as an early PSII assembly factor
Source: Plant Cell. 2025 Mar 17;37(6):koaf055. doi: 10.1093/plcell/koaf055 (PMC12164590; doi:10.1093/plcell/koaf055)
Supplement: koaf055_Supplementary_Data [file koaf055_supplementary_data.zip › psb28_interactive.zip!/plots/intensity/qualityPlots/peptides/_Cre05.g230850.t1.1.html]

### Cre05.g230850.t1.1

Trivial name:   
SeqCoverage [median]: 32.70  
Mass: 60.65 kDa  
  
 

| Sequence | MissedCleavage | Mass [Da] | wtIntensity | muIntensity | Score |
| --- | --- | --- | --- | --- | --- |
| AAAEAVSAVK | 0 | 915.50 | 1401.04 | 571.94 | 78.82 |
| AAAELGSK | 0 | 745.40 | 288.04 | 0.00 | 84.50 |
| AAAGEAAKPVAEAAK | 1 | 1353.73 | 3792.55 | 1905.18 | 74.79 |
| AEPHSPELLELAATLYK | 0 | 1880.99 | 478.80 | 0.00 | 42.81 |
| AVAEASNAAAGAAAATGQAATTAAANVTK | 0 | 2500.25 | 605.40 | 0.00 | 40.46 |
| AVGSAATDASR | 0 | 1004.49 | 2441.83 | 873.82 | 131.45 |
| GVAAPEPAAAR | 0 | 1008.54 | 634.36 | 0.00 | 76.22 |
| LGAAAAIK | 0 | 713.44 | 783.76 | 0.00 | 88.73 |
| SEGVGEQR | 0 | 860.40 | 609.10 | 0.00 | 87.18 |
| TAVAEAPK | 0 | 785.43 | 286.97 | 0.00 | 101.60 |
| VAAGAVEAAGAAHAAADK | 0 | 1549.78 | 3450.97 | 947.43 | 105.28 |
| VAEAAAAGAAHAAAAGGNAVK | 0 | 1747.90 | 4250.94 | 2712.90 | 84.03 |
| VVAAQAAAAKPAAEK | 1 | 1394.79 | 2990.34 | 366.49 | 84.57 |
|  |  |  | 22014.1 | 7377.8 |  |
